# Treatment outcomes and the associated factors among breast cancer patients in Tanzania: a retrospective cohort study

**DOI:** 10.3332/ecancer.2025.1874

**Published:** 2025-03-18

**Authors:** Nashivai E Kivuyo, Daniel W Kitua, Martin E Chikelea, Mungeni A Misidai, Ally H Mwanga, Obadia V Nyongole, Larry O Akoko

**Affiliations:** Department of Surgery, Muhimbili University of Health and Allied Sciences, Dar es Salaam 65001, Tanzania

**Keywords:** breast cancer, breast malignancy, %-year outcomes, breast cancer in Africa, breast cancer survival, treatment outcomes

## Abstract

**Background:**

Breast cancer (BC) is the second most prevalent cancer among women in sub-Saharan Africa. Despite dedicated efforts to enhance BC care in the region through improving diagnostic and treatment services, little is known about the treatment outcomes of BC patients and the predictors of outcomes in our local settings have not been enumerated. This study aimed to investigate the treatment outcomes and the associated factors among BC patients in Tanzania.

**Materials and methods:**

This was a retrospective cohort study at Muhimbili National Hospital and Ocean Road Cancer Institute in 2022. It involved female patients 18 years and above who were confirmed to have BC by histology. A sample size of 240 was determined to be adequate to detect a survival difference between the stages. Using Research Electronic Data Capture, clinical characteristics were collected from patients’ treatment records and survival status was ascertained both by case notes or phone calls to patients or next of kin. Data were transferred into Statistical Package for the Social Sciences version 27 for subsequent analysis where continuous variables were summarised as proportions. We used chi-square and Fisher’s exact tests to determine the association between various patients’ characteristics and treatment outcomes. Kaplan-Meyer analysis was used to determine survival and a p-value less than 0.05 is considered statistically significant.

**Results:**

In total, 298 BC patients were studied with a mean age of 53.2 ± 13.6 (27–89). Invasive ductal carcinoma, parity and late stage at presentation were predominant features in these patients. A triple negative subtype was identified in 35.2% of the women. Only 27.9% and 33.6% of the patients received neoadjuvant and adjuvant chemotherapy respectively, while 8.1% of the patients were palliated. The overall 5 years survival was 29.7%, while being significantly poor in patients with advanced stages of the disease. Luminal subtypes, parity, menopausal status and age had some influence on BC survival among our patients but not in a significant manner.

**Conclusion:**

Mastectomy is predominantly offered to BC patients in Tanzania with no standardisation of use of chemo/radiation both in neo/adjuvant settings. Some important prognostic factors were missing including a lack of standardised work up of patients. With the predominance of advanced stage at presentation, BC carries unacceptable high mortality in Tanzania. Efforts to detect BC early, understand patients’ perception of their disease and standardisation of care are needed to successfully implement treatment guidelines.

## Background

Breast cancer (BC) is the most prevalent cancer among women in sub-Saharan Africa (SSA) [1]. Studies predict that the incidence of BC in the region is expected to double by 2040 owing to population aging and lifestyle changes [2]. In Tanzania, BC is the second most prevalent cancer among females, following cancer of the cervix [3]. The age-standardised incidence rate of BC in the country is approximately 19.4 per 100,000 women [4]. Notably, BC accounted for 4,000 new cancer cases and approximately 2,000 cancer-related deaths in Tanzania in 2020 alone [5]. This makes BC a significant public health concern in the country.

Globally, significant progress has been made in advancing BC care with a focus on enhancing overall clinical outcomes [6]. This is also evident in SSA, where most countries, including Tanzania, have established various initiatives to improve BC control and enhance BC care [3]. These efforts encompass impactful campaigns designed to raise community awareness, along with dedicated initiatives aimed at increasing accessibility to diagnostic and treatment services [7, 8]. However, there is a paucity of data regarding the treatment outcomes of BC patients in Tanzania.

Studies have highlighted geographic variations in BC treatment outcomes globally [9]. This variation is attributed not only to disparities in diagnostic and treatment advancements but also to variations in the patients’ demographics, presentation and tumour biology [10]. Additionally, studies from high-income countries (HICs) suggest that BC is more aggressive in black women, leading to suboptimal survival even with optimal treatment [10, 11]. Given the paramount emphasis of utilising local evidence to guide interventions for cancer control in SSA [13], a comprehensive understanding of the treatment landscape and outcomes for BC patients in Tanzania is essential, hence this study.

## Methods

### Study design and setting

This retrospective cohort study involved female patients with a histologically confirmed diagnosis of BC who were treated at Muhimbili National Hospital (MNH) and the Ocean Road Cancer Institute (ORCI), both located in Dar es Salaam, Tanzania. These hospitals act as tertiary referral centers for the entire country, which has a population of approximately 60 million people, particularly for cancer care. As teaching hospitals affiliated with Muhimbili University of Health and Allied Sciences, both institutions have the capability to diagnose (through histopathology and imaging), treat (via mastectomy, chemotherapy and radiotherapy), and follow up with patients diagnosed with BC. The patient population at these hospitals comes from all regions of the country, providing a representative perspective on BC care in Tanzania. During their treatment, imaging modalities for detecting pulmonary metastasis included X-rays and computed tomography (CT) scans, while abdominal ultrasounds or CT scans were utilised for identifying visceral metastasis.

### Study population and sample

To be included in the study, patients had to be 18 years or older at the time of treatment and possess histological evidence of BC from a histopathology report at MNH, signed by at least two pathologists. Additionally, patients needed to have completed a minimum of 5 years of follow-up since their diagnosis, meaning they must have been treated between January 2016 and December 2017. Patients with missing records were excluded from specific components of the analysis. To detect a survival difference of at least 20% between patients with locally advanced disease and those with metastatic disease, the study aimed for a power of 80% and a two-tailed alpha level of 0.05. This required at least 240 patients. However, with 256 eligible patients meeting the inclusion criteria, the study’s power increased to 85.5%.

### Identification of study subjects, variables and data collection

Patients were initially identified using a cancer diagnosis registry maintained at the MNH histopathology laboratory, which is part of the multidisciplinary tumour board required for any cancer-directed therapy at both hospitals. From this registry, we obtained the names, hospital registration numbers and final pathological diagnoses, which were compiled into an Excel spreadsheet. Using this information, we retrieved individual electronic histopathology reports and case notes from MNH and ORCI to extract clinicodemographic details. The independent variables recorded included the patient’s age (calculated from the date of birth), the documented clinical stage of the disease, tumour histological type, hormonal status (progesterone receptor, estrogen receptor) and human epidermal growth factor receptor 2 (HER2/neu) status. These details were obtained from the signed histology reports from the central pathology laboratory. Additional variables included the treatments offered. The same identification process was conducted at ORCI to gather data on the oncology-directed care provided to each patient. The last documented status was considered final if the patient was deceased. For those listed as alive, we used the available contact information to reach out to patients or their designated next of kin to confirm their current status. The study endpoint was defined as 5 years after the date of histological confirmation of BC, at which point survival status was documented as either alive or deceased. Data collection was conducted using Research Electronic Data Capture (REDCap).

### Data analysis

After checking for completeness, patient identifiers such as names and registration numbers were removed to deidentify the information. The data were then transferred from REDCap to the Statistical Package for the Social Sciences (SPSS) version 27 for analysis. Categorical variables were summarised as frequencies and proportions, while continuous variables were summarised as measures of central tendency. To determine associations between independent and dependent variables, chi-square tests and Fisher’s exact tests were conducted. Additionally, Kaplan-Meier survival analysis curves were used to compare survival differences, also with a significance level set at *p* < 0.05.

### Ethics approval

The study protocol was reviewed and approved by the Institutional Review Board of MNH with approval number MNH/IRB/I/052. No direct patient identifiers were used during data analysis following the deidentification process. Direct identifiers collected during data abstraction were removed before transferring the data into SPSS software to maintain patient anonymity. The study adhered to the Helsinki Principles for the conduct of research involving human subjects.

## Results

### Recruitment of study participants

We identified a total of 354 records of BC patients treated at MNH between January 2016–December 2017 from the CPL. We excluded 56 patients due to missing case notes and hence could not obtain the study variables other than a BC diagnosis. The clinicodemographic characteristics of the remaining 298 patients were analysed and presented below. In the survival analyses, we excluded 42 more patients due to failure to ascertain survival status despite efforts of calling the patient and the next of kin.

### Description of study participants

In Table 1 below, we present a summary of the clinical demography of the study participants. The mean age of women with BC was 53.2 ± 13.6 (27–89) years with the majority being in the age group 41–60 years by 129 (51.7%) while 56 (22.8%) were under 40 years of age. Parity status was available for only 228 (76.5%) women where almost all were parous at 220 (96.5%) while menopausal status was nearly equally distributed among 156 (52.3%) women in which the information was available, 77 (49.4%) and 79 (50.6%) for pre- and post-menopausal respectively. Most patients presented with locally advanced disease in stage III with the majority of them being in stage IIIB 187 (62.8%) while only 18 (6.0%) and 50 (16.8%) had stage IIB and IV, respectively. Most BCs were located on the right side 158 (53%). The most common histological type was ductal carcinoma, found in 278 (93.2%) patients, followed by medullary carcinoma in 16 (5.4%). Immunohistochemistry results were available for only 159 (53.4%) patients, among which Luminal A was the most predominant subtype, present in 68 (42.7%) cases, followed by TNBC in 56 (35.2%), with luminal B and HER2-enriched being the least common subtypes, at 21 (13.2%) and 14 (8.8%), respectively.

### Treatment modalities of BC patients

**Figure 1.**
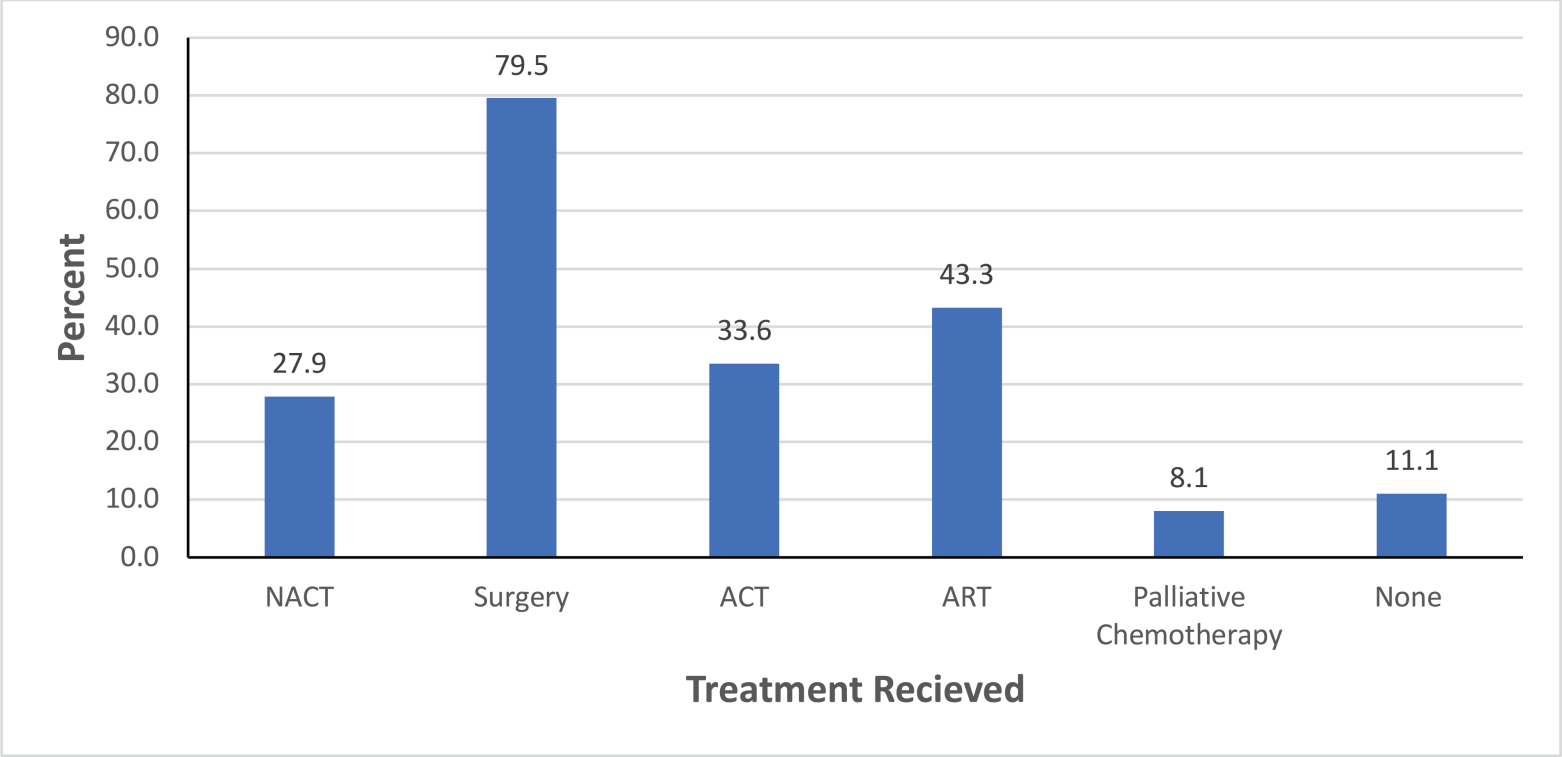
A bar graph showing treatment modalities among BC patients attended at MNH/ORCI between January 2016 and December 2017. NACT, Neoadjuvant chemotherapy; ACT, Adjuvant chemotherapy; ART, Adjuvant radiotherapy.

In Figure 1 below, we display a bar chart of the treatment landscape among the 298 BC patients. Surgery was the most common treatment modality, with 237 patients (79.5%) undergoing this procedure. The only form of neoadjuvant therapy administered was neoadjuvant chemotherapy (NACT) which was given in 76 patients. Following surgery, 100 patients (33.6%) received adjuvant chemotherapy (ACT) and 129 patients (43.3%) were treated with adjuvant radiotherapy (ART). 24 patients (8.1%) received palliative chemotherapy and 33 patients (11.1%) did not receive any cancer-directed treatment.

### Treatment modalities of BC patients according to stage

**Figure 2.**
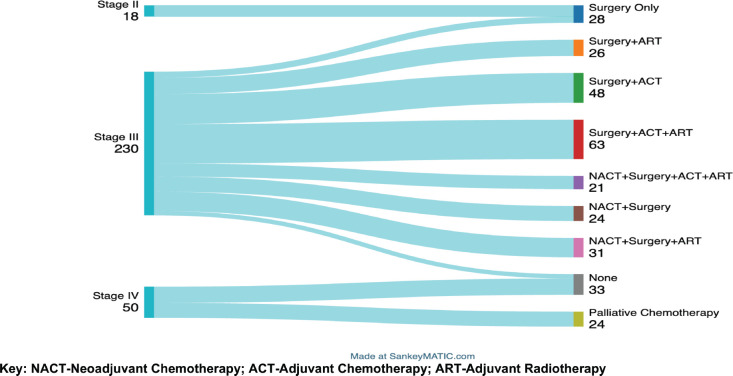
A Sankey diagram showing treatment modalities according to disease stage among the BC patients attended at MNH/ORCI between January 2016 and December 2017. Key: NACT, Neoadjuvant chemotherapy; ACT, Adjuvant chemotherapy; ART, Adjuvant radiotherapy.

The Sankey diagram in Figure 2 below displays the treatments offered by stage of disease grouped as stages II–IV. All patients with stage II received only surgery (mastectomy) with no additional treatment for their cancer. In Stage III, treatment modalities varied among the 230 patients: all underwent a mastectomy, with 147 (63.9%) having upfront surgery and 76 (33%) receiving NACT before surgery. A majority of stage III patients,189 (82.2%), received adjuvant therapy, with 120 (52.2%) receiving ART and 69 (30%) receiving ACT. Of the 50 patients with metastatic disease, 24 were palliated by chemotherapy while 26 received no cancer-directed therapy.

### Overall 5-year survival of BC patients

**Figure 3.**
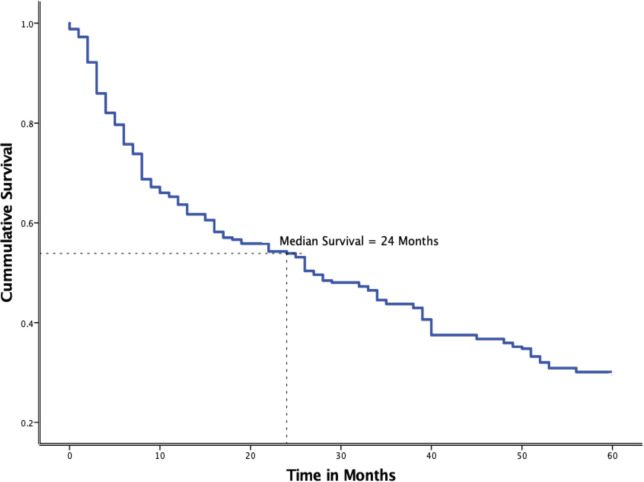
Kaplan-Meier plot showing overall survival among 256 BC patients treated at MNH/ORCI in 2016 and 2017.

Figure 3 displays the Kaplan-Meier 5-year survival probability of the 256 BC patients whose survival status could be ascertained. The overall median survival of BC patients in Tanzania is 24 months. By the end of year 1, only 66.4% of the patients were alive, the sharpest yearly drop in this cohort. The deaths steadied but did not plateau in the subsequent years with a yearly drop of 10.9%, 11.4%, 7.8% and 6.6% by the end of 5 years, respectively. Only 76 (29.7%) patients were alive at the end of 5 years of follow-up.

### Association between age, stage of disease, parity, menopausal status and luminal status and 5 years survival status

Table 2 below shows the association between age and survival, with older age groups showing higher survival. Patients less than 40 years of age had the lowest survival compared to other age groups but this was not statistically significant, *p*-value 0.25. Early stages, IIB and IIIA, demonstrated higher survival (62.5% and 56.0%, respectively), compared to the advanced stages (IIIB-C and IV) a finding that was statistically significant, *p*-value <0.001. Luminal A and B had higher survival probability when compared to Her2-enriched and TNBC subtypes but this difference failed to reach statistically significant levels, *p*-value 0.07. Parous and postmenopausal women had higher survival compared to nulliparous and premenopausal, respectively, but these was not statistically significant.

### Desegregating the 5-year survival by stage at diagnosis

**Figure 4.**
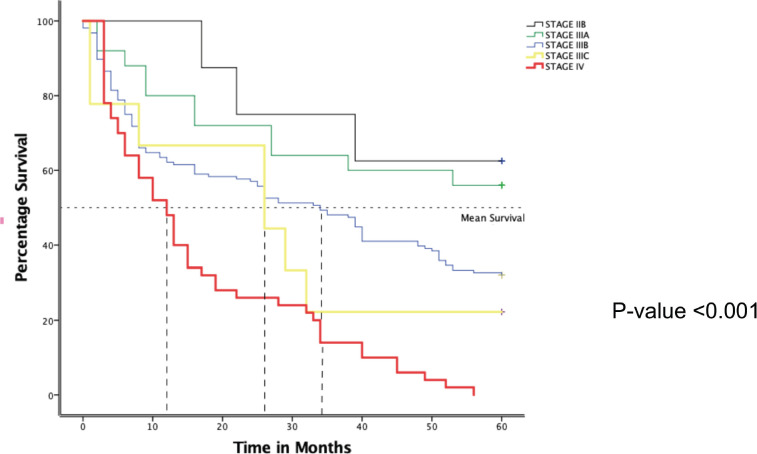
Kaplan-Meier plot showing overall 5-year survival of BC patients attended at MNH from January 2016–December 2017 according to stage.

In Figure 4 below, the Kaplan-Meier curve depicts varied 5-year survival between the different stages among 256 BC women attended at MNH/ORCI. By the end of the first year, only half of stage four patients were still alive compared to about 60% (Stage IIIC and B), 80% Stage IIIA and 100% Stage IIB. A progressive drop in survival continued for patients with stage IV with none surviving at the end of the 5 years follow up period. A steep drop was observed among stage IIIC patients at 3 years which later steadied with a second drop at 4 years, and only 11% surviving at 5 years. Patients with stage IIIB also witnessed a progressive drop in survival throughout but about 32.9% were still alive at the end of 5 years. Patients with stage IIIA observed drops with periods of plateau that steadied after the first year with 56.0% alive at the end of 5 years. Stage IIB patients witnessed a longer plateau with the sharpest drops in the later parts of the first 2 years. At the end of the follow up period, 62.5% were still alive with no death observed during the last 2 years. This observed survival difference was statistically significant, *p*-value <0.001.

### Desegregating the 5-year survival by luminal sub-type

**Figure 5.**
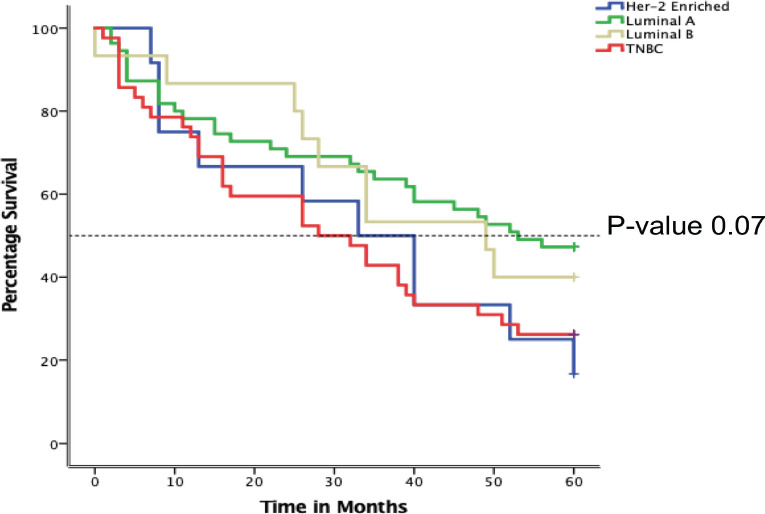
Kaplan-Meier plot showing overall5-year survival of BC patients attended at MNH from January 2016–December 2017 according to luminal sub-types.

In Figure 5 below, the Kaplan-Meier curve depicts varied 5-year survival between the different luminal subtypes among 159 BC women attended at MNH/ORCI. TNBC displays the lowest survival throughout the time period, with a mean survival of 28 months (12.6–43.4). This is followed by Her-2 enriched with mean survival of 33 months (17.2–48.8). Luminal subtypes demonstrated overall better survival at 49 and 51 months, respectively. The difference in survival by BC subtypes was, however, not significant, *p*-value of 0.07.

## Discussion

In this study, we describe the treatment landscape and 5-year outcomes of BC from two leading cancer treatment hospitals in Tanzania. We believe this is the first study conducted since the introduction of a national cancer treatment guideline harmonised with the NCCN SSA guidelines. By discussing key points from our findings, we aim to reflect on the existing landscape, which will help assess the implementation status of the new guidelines and their impact on patient survival within the Tanzanian context.

While this study was not designed to establish the epidemiology of BC in Tanzania, being hospital-based in nature, it provides insights into the demographics of patients seeking treatment. More than half of our patients were between 40 and 60 years of age, which aligns with existing literature on current age trends in BC [14, 15]. The predominance of BC in this age group in Tanzania has remained stable since 2010 [16]. Notably, 1 in 5 treated patients were under 40 years of age, indicating a need for further investigation into the potential genetic contributions to BC among Tanzanian women contributing to early onset BC. Menopausal status was documented for just over half of the patients, and some had no information regarding parity status. Although not fully characterised, BC was found to be most common among parous women, which contradicts findings typically observed in Western BC populations [16].

Ductal carcinoma not otherwise specified (NOS) was the most prevalent diagnosis among women with BC, documented in 90% of the patients treated at our facilities. This finding is consistent with reports from the region [11, 17]. However, further characterisation of histology is limited, making it challenging to perform subgroup analyses on treatment outcomes using additional variables. In this study, luminal subtypes were reported in less than half of the patients, and tumour grade reporting was incomplete. Given the crucial role of immunohistochemistry and tumour grade analysis in treatment decision-making and prognostication [18], there is a compelling need to investigate the factors that hinder their routine evaluation in our setting.

The distribution of disease staging revealed a significant prevalence of locally advanced and metastatic disease, which may be attributed to delays in presentation, aggressive tumour biology or a combination of both. This requires further investigation, and a qualitative study may be necessary to better understand the delays involved. Other research suggests that these observations are consistent with delays in presentation [19]. Potential factors contributing to these delays may include a lack of awareness about the disease, a high concentration of the rural population and limited access to vital health information [3, 20, 21]. These findings underscore the urgent need for targeted awareness campaigns, improved healthcare infrastructure and enhanced health education efforts to address the challenges associated with timely diagnosis. Ultimately, these efforts aim to mitigate the impact of BC within underserved populations.

In about 1 in 10 of the cases, no cancer-directed treatment was offered, likely reflecting late-stage presentation alongside poor performance or nutritional status. Of particular concern in this cohort were the seven patients with stage III disease who did not receive treatment and subsequently succumbed to stage IV disease. This finding mirrors a report from Northwestern Tanzania, where over 20% of patients did not receive any cancer-directed therapy after being diagnosed with BC at a treatable stage [22]. Understanding patients’ perspectives regarding BC diagnosis and treatment is crucial in the local context to improve therapy uptake and survival rates.

Neoadjuvant therapy was administered to only 1 in 3 patients with clinical stage III disease. While there is ongoing debate about the necessity of NACT for all stage III cases, its benefits remain significant, as it can help identify patients whose cancer responds to treatment and who would benefit from its administration. Additionally, some patients did not receive adjuvant therapy after surgery. Various factors, both patient-related and healthcare-related, influence this outcome [23]. The Tanzanian BC treatment guidelines clearly define the roles of neoadjuvant and adjuvant therapy for patients with BC [24]. There is, therefore, a compelling need for dedicated advocacy to ensure that BC treatments align with the recommended guidelines.

BC in Tanzania is highly lethal, as demonstrated by very low overall survival rates compared to existing literature [12, 25, 26]. Less than 3 of 10 of BC patients were alive at the end of the 5-year follow-up period. Several factors affect BC survival, some of which were not fully captured in this study. The clinical stage is one of the most important determinants of 5-year survival. Therefore, accurate staging is essential, as some patients diagnosed as clinical stage III may have had metastatic disease at the time of treatment. The lack of FDG PET scans and the underutilisation of bone scans – which are available at ORCI – could have improved clinical staging. Most patients at this hospital receive chest X-rays and abdominal ultrasounds instead of CT scans, which are more effective in detecting metastases. This could explain the initial steep survival curve among stage IIIC patients compared to stage IV patients.

Another factor influencing survival outcomes is the adoption of standardised and personalised treatment. This approach not only depends on the clinical stage but also considers the tumour biology of the patients, allowing for tailored treatments even among those with similar stages at presentation. While tumour biology did not show a significant effect on survival, TNBC was associated with the worst outcomes. Among patients with luminal status, about one in three had TNBC, a finding that aligns with regional data but is much higher than reports from Western countries [11]. This discrepancy in luminal subtypes has important implications for management and prognosis, as TNBC is known to be associated with poorer outcomes [23, 24].

We were unable to determine whether any targeted therapies were offered to patients due to a significant number lacking luminal status, making it difficult to confidently establish the true effect of luminal status on survival. Additionally, some patients may experience early death due to toxicity from cancer-directed therapies [27], which was not studied due to the lack of standardised treatment and clear cause of death documentation. Future implementation studies should collect robust data that will allow assessment of the effect of various factors on BC survival.

For the new guidelines to improve BC survival in Tanzania, an implementation strategy involving multidisciplinary healthcare providers across the cancer care continuum is required to ensure standardised care from diagnosis and staging to treatment. Although this study is the first of its kind to report treatment outcomes among BC patients, it has limitations, including missing data and potential inconsistencies due to its retrospective design. The diagnosis of BC at advanced stages remains a persistent problem in Tanzania, likely due to a lack of screening services and overall capacity. While local guidelines exist to promote early detection, these are still in their infancy and the impact has not yet been realised. Furthermore, comprehensive treatment approaches for BC patients, which combine neoadjuvant treatment, surgery and adjuvant therapy, are needed to enhance therapeutic outcomes.

## Conclusion

BC patients in Tanzania present late with locally advanced or metastatic diseases. Most are diagnosed in the fifth decade of life. There is a relatively higher proportion of TNBC compared to that reported in Western literature. Overall survival is very low compared to HICs but similar to other SSA. Late stage at diagnosis is significantly associated with poor 5-year survival.

## Conflicts of interest

No conflicts of interest to declare.

## Data availability

The data that support the findings of this study are available from the corresponding author upon reasonable request.

## Ethical statement

The authors are accountable for all aspects of the work in ensuring that questions related to the accuracy or integrity of any part of the work are appropriately investigated and resolved. MNH IRB approved this study in accordance with the declaration of Helsinki. The IRB number is MNH/IRB/I/052.

## Author contributions

(I) Conception and design: All authors (II) Administrative support: AH Mwanga, OV Nyongole, LO Akoko; (III) Provision of study materials or patients: None; (III) Collection and assembly of data: NE Kivuyo, DW Kitua, ME Chikelea; (IV) Data analysis and interpretation: All authors; (V) Manuscript writing: All authors; (VI) Final approval of manuscript: All authors.

## Figures and Tables

**Table 1.** Showing clinical demography of women treated for BC between 2016 and 2017 at MNH/ORCI in Tanzania (*n* = 298).

Description	Frequency (%)
Age	
<40	68 (22.8)
41–60	154 (51.7)
61–80	62 (20.8)
>80	14 (4.7)
Histology	
Ductal carcinoma NOS*	278 (93.2)
Medullary	16 (5.4)
Others	4 (1.3)
Disease stage	
Stage IIB	18 (6.0)
Stage IIIA	34 (11.4)
Stage IIIB	187 (62.8)
Stage IIIC	9 (3.0)
Stage IV	50 (16.8)
Laterality	
Right	158 (53.0)
Left	140 (47.0)
Parity (*N* = 228)	
Nulliparous	8 (3.5)
Parous	220 (96.5)
Luminal status (*N* = 159)	
Luminal A	68 (42.7)
Triple negative	56 (35.2)
Luminal B	21 (13.2)
Her2 enriched	14 (8.8)
Menopausal status (*N* = 156)	
Premenopausal	77 (49.4)
Post-menopausal	79 (50.6)

* NOS- Not Otherwise Specified

**Table 2.** Shows the relationship between selected patients’ demography and 5-year overall survival among women with BC attended at MNH/ORCI between 2016 and 2017.

Variable	Alive	Dead	*p*-value
Age groups (*N* = 256)			
<40	11 (19.6)	45 (80.4)	0.25
41–60	40 (31.0)	89 (69.0)	
61–80	21 (35.6)	38 (64.4)	
>80	4 (33.3)	8 (66.7)	
Disease stage (*N* = 256)			
IIB	10 (62.5)	6 (37.5)	< 0.001
IIIA	14 (56.0)	11 (44)	
IIIB	50 (32.1)	104 (67.9)	
IIIC	2 (22.2)	7 (77.8)	
IV	0 (0.0)	50 (100)	
Parity (*N* = 198)			
Nulliparous	51 (26.6)	141 (73.4)	0.71
Parous	2 (33.3)	4 (66.7)	
Menopausal status (*N* = 134)			
Premenopausal	16 (24.2)	50 (75.8)	0.16
Postmenopausal	24 (35.3)	44 (64.7)	
Luminal status (*N* = 124)			
Luminal A	26 (47.3)	29 (52.7)	0.07
Luminal B	6 (40.0)	9 (60.0)	
Her-2 enriched	2 (16.7)	10 (83.3)	
Triple Negative	11 (26.2)	31 (73.8)	
